# Impact of Multimedia Messaging Service Education and Exercise Social Support on Physical Activity Among Patients With Type 2 Diabetes: Quasi-Experimental Study

**DOI:** 10.2196/42590

**Published:** 2023-05-22

**Authors:** Mohammad S Alyahya, Nihaya A Al-Sheyab, Yousef S Khader, Jumana A Alqudah

**Affiliations:** 1 Health Management and Policy Faulty of Medicine Jordan University of Science and Technology (JUST) Irbid Jordan; 2 Allied Medical Sciences Department/Faculty of Applied Medical Sciences Jordan University of Science and Technology (JUST) Irbid Jordan; 3 Department of Public Health and Community Medicine Jordan University of Science and Technology (JUST) Irbid Jordan

**Keywords:** diabetes mellitus, educational interventions, multimedia messaging service, physical activity, social support

## Abstract

**Background:**

Social support is one of the interpersonal stimuli that define an individual’s predisposition to engage in health-promoting behaviors and is considered a facilitator in improving health habits. Patients with type 2 diabetes mellitus (T2DM) can benefit from educating supportive families and friends on self-care management including exercise behavior. Multimedia messaging service (MMS) could also be an effective method for delivering targeted educational interventions that focus on physical activity (PA).

**Objective:**

This study aimed to assess the effectiveness of MMS educational interventions and perceived social support for exercise on level of PA of patients with T2DM.

**Methods:**

A quasi-experimental pretest-posttest design was conducted to recruit 98 patients with T2DM. The intervention group received MMS education aiming to improve exercise social support and PA level for 2 months, and their counterparts in the control group received the usual routine care. We sent 2 to 3 messages daily for 2 weeks from Saturday to Thursday (12 days total). These messages were a combination of videos and texts, and the evidence-based content of these messages was reviewed and approved by the advisory committee. We randomly assigned eligible patients in a 1:1 ratio into the intervention or the control groups. Participants completed a survey in 3 periods.

**Results:**

There were no significant differences in friends’ support, family verbal, practical, or emotional support over time in the intervention group (*P*>.05). Yet, there was a small effect size (Cohen *d*) in friends’ social support (0.389), family practical support (0.271), and moderate activities (0.386). A medium effect size was found in family verbal (0.463) and emotional (0.468) support. Being married increased the likelihood of friends’ support by 2.3 times after intervention (*P*=.04), whereas rarely doing exercise decreased the likelihood of friends’ support by 28% (*P*=.03) and family practical support by 28% (*P*=.01). Being female and married increased the likelihood of doing moderate activities by 1.6 times (*P*=.002) and 1.5 times (*P*=.049) in the intervention group. Being a housewife decreased the likelihood of doing moderate activities by 20% (*P*=.001). Finally, being a female with a higher educational level decreased the likelihood of doing hard activities by 20% (*P*=.04) and 15% (*P*=.002), respectively.

**Conclusions:**

A theoretically based MMS health education targeting PA levels and social support of family and friends to perform PA seems promising in promoting family and friends’ social support and improving PA levels among patients with T2DM. Actively involving family and friends in educational interventions that target PA can have an impact on health-promoting behaviors in patients with diabetes.

## Introduction

The prevalence of type 2 diabetes mellitus (T2DM) is mounting in low- and middle-income countries at a higher rate than that in developed countries due to several factors including unhealthy diet, aging, obesity, and sedentary behaviors [1-4]. A recent national study reported that the prevalence rate of diabetes mellitus in Jordan was 32% in men aged older than 25 years and 18% in women [5], and is expected to increase even more as a result of escalating obesity rates, physical inactivity, and dietary pattern changes [5-7]. This is alarming as the consequences of uncontrolled and poorly managed diabetes mellitus can influence both national and global economics [1,4,8,9].

Yet, several self-management educational interventions showed effectiveness in improving overall health for patients with T2DM [4,10,11]. These interventions focus on the adoption of specific lifestyle behaviors, such as healthy nutrition, weight management, and regular physical activity (PA) [7,10,12-14]. For instance, performing regular PA can delay diabetes complications by enhancing insulin sensitivity as well as insulin receptors [13,15]. Despite all benefits of PA, patients with T2DM in Jordan are physically inactive and, therefore, need to be encouraged to engage in regular PA and improve their adherence through clinical care and media education [7].

Social support is one of the interpersonal stimuli that define an individual’s predisposition to engage in health-promoting behaviors and is considered a facilitator in improving health habits [16,17]. Social support either from family members or friends is beneficial in adopting regular PA [18,19]. Patients with T2DM can benefit from educating supportive families on self-care management [20]. In specific, exercise social support is essential in educational interventions that focus on improving PA for patients with T2DM, who usually receive less support for PA than that for diabetes management medication and diet [21]. This is particularly important to consider when designing educational interventions as patients with T2DM who received social support from family members showed positive diabetes self-management practices [22]. Nonetheless, social support alone may not be enough when educating patients with T2DM [23] highlighting the need to incorporate other concepts besides social support while designing educational interventions to promote PA, such as exercise self-efficacy and perceived exercise benefits and barriers [7].

One approach that can help in supporting the self-management of patients with T2DM is information and communication technologies, such as mobile technology, which is convenient and useful in accessing a broader population [4,24-26] and can improve the quality of care for patients with T2DM, such as SMS text messaging [27,28]. In Jordan, cell phones are commonly used and widely available [29,30]. Multimedia messaging service (MMS) may be an appropriate method for delivering targeted educational interventions. Educating patients with T2DM through MMS could be beneficial in promoting self-management behaviors [31]. Thus, this study aimed to assess the effectiveness of a theoretically based distant MMS-based educational intervention and perceived social support for exercise on the level of PA of patients with T2DM.

## Methods

### Design and Setting

A pretest-posttest, quasi-experimental design was carried out over 2 months (June-October 2020) at the Family Medicine, Endocrinology, and Diabetes clinics at a leading university hospital (King Abdullah University Hospital) and affiliated outpatient clinics in the North of Jordan.

### Sampling Process

A nonprobability convenience sampling method was used to recruit 100 patients with T2DM at baseline. During the first (1 month after baseline data collection) and the second (2 months after baseline data collection) follow-up period, 1 patient from each group dropped out, totaling 98 (49 in the intervention group and 49 in the control group). Patients were recruited while they were waiting for their appointment in the identified clinics. Inclusion criteria were the capacity to perform PA, the ability to read and write, owning a smartphone, and the ability to walk without a cane. We randomly assigned eligible patients in a 1:1 ratio into the intervention or the control groups. Patients in the intervention group received the MMS educational messages and their counterparts in the control group received the usual routine care.

### Recruitment Process

The recruitment process was initiated and eligible patients with T2DM were voluntarily invited to participate when they attended the clinics based on a previous appointment. Those who agreed to participate were asked to sign an informed consent and complete a survey. Then, they were randomly assigned into two groups based on the sequential order; one in the intervention group and the next in the control group until the final sample size was obtained. The first follow**-**up was conducted one month after baseline data collection using telephone interviews to fill out the survey at follow-up. The second follow-up was undertaken 2 months after the baseline data assessment using telephone interviews also to fill out the survey for the third time.

### Instruments

A 2-part survey was used in this study. The first part included information about participant’s sociodemographic characteristics (eg, age, sex, education, occupational status, and BMI) as well as type of medication, previous related behaviors, and blood sugar level. The second part consisted of following two questionnaires as below:

1. Perceived social support scale for exercise behavior [32,33]. This 5-point Likert scale was used to evaluate participants’ perceptions of family support (15 items) and friends support (5 items).
2. A 7-day physical activity recall questionnaire [34]. Patients were asked to determine the duration (in minutes) and type of each physical activity (daily activities or leisure activities) to estimate total energy expenditure over 7 days. There were 3 separate assessments designed to ascertain participant’s pattern of PA. A series of closed-ended questions were used to estimate participation in moderate activities, challenging activities, or vigorous activities.

### The Intervention

Participants in the MMS group (intervention group) received educational messages based on the Health Promotion Model [16]. We sent 2 to 3 messages daily for 2 weeks from Saturday to Thursday (12 days total). These messages were a combination of videos and texts, and the evidence-based content of these messages was reviewed and approved by the advisory committee. Each of the 12 days has customized messages and videos about the benefits of PA on patients with T2DM—including mental benefits and its effect on blood sugar (increased perceived benefits); appropriate way to perform PA (before, during, and after) (reduction of perceived barriers); appropriate duration and types of PA for patients with T2DM (increased perceived self-efficacy); information on why blood glucose increases during or after PA and how to care for and prevent foot ulcers (reduced perceived barriers); recommendations to integrate PA in daily life and how PA’s commitment and motivation increase with a buddy partner (increased perceived benefits and increased social support); and guidance to assure patients to ask support from friends and family in care and accompaniment (perceived social support).

After 2 weeks of daily educational messages through MMS, the intervention group participants received 2 messages weekly for up to a month and a half. During this time, we stayed in close contact with patients in case of any questions or complaints and to ensure that they are in good health. Participants in the intervention group also kept communicating back with the research team to show satisfaction with being involved in the study.

### Data Analysis

SPSS software (version 22; IBM Corp) was used to analyze the data. Descriptive analysis was first carried out to present the frequencies and percentages of demographics and health status in both groups. The normality of data was also tested, and nonparametric tests were performed whenever the normality assumption was violated. Then, the Mann-Whitney *U* test and chi-square tests were performed to determine if there was a significant difference between the groups in baseline data. A comparison between baseline data, first, follow-up data, and second follow-up data according to perceived health status scale and exercise social support and PA recall scales was conducted to determine if there was a significant difference between the 3 data sets in these subscales. We also compared baseline data with the second follow-up of the intervention group on exercise social support and PA recall scales to calculate the effect size using the Cohen *d* value. Finally, logistic regression analysis of social support and PA recall scales with demographics and health status was also performed to determine the variables that significantly affect social support and PA recall scales. The significance level was set at *P*<.05.

### Ethics Approval

Before data collection, ethical approval was obtained from the Jordan University of Science and Technology institutional review board (35/130/2020; March 8, 2020). Patients with DM who were willing to participate in the study were invited to sign a consent form in Arabic, including a statement informing them that he or she had the right decline participation at any time. The data were treated anonymously and confidentially. Furthermore, a number of considerations were taken into account to ensure the safety of participants. These considerations were printed out and attached to the consent form, including the following:

(1) If the patients notice a sudden rise in the level of sugar in the blood after entering the study, they should stop doing physical activities and visit their doctor or call him or her and explain their condition as soon as possible.

(2) Keep an eye on your blood sugar levels and check them frequently before and after exercise, especially if you are new to exercise.

(3) Do not exercise if your blood sugar levels are too low or too high. If they are lower than 100 mg/dL or higher than 250-300 mg/dL, it may not be safe to work out; so, eat a snack or wait for it to reach a better level before getting started.

(4) Stop exercising if you experience dizziness, shortness of breath, disorientation, or pain. 

(5) When the patient goes out to exercise in the gym or to take a walk or any other outdoor activity, he or she should take at least 15 gm of sugar (fruits, juice, and candy) in anticipation of a low level of sugar in the blood while the patient is far from home.

## Results

### Sociodemographic Characteristics

Sociodemographic characteristics of participants are presented in Table 1. There was no statistically significant difference between the intervention and control groups in terms of sociodemographics and perceived health status variables at baseline. The majority of participants were female, aged 55-64 years, married, and housewives in both groups. A total of 21 (42%) participants in the intervention group have a secondary level of education and 21 (42%) have bachelor’s degrees.

**Table 1:** Sociodemographic characteristics of participants with type 2 diabetes mellitus in the 2 study groups at baseline.

Variables		Intervention group (N=50), n (%)		Control group (N=50), n (%)		*P* value
**Age (years)**						.16^a^
	25-34	3 (6)	2 (4)			
	35-44	5 (10)	3 (6)			
	45-54	16 (32)	13 (26)			
	55-64	22 (44)	25 (50)			
	65-74	4 (8)	7 (14)			
Female sex		38 (76)		34 (68)		.37
**Marital status**						.94
	Single	1 (2)	1 (2)			
	Married	44 (88)	45 (90)			
	Widower	5 (10)	4 (8)			
**Educational level**						.53^a^
	Primary	6 (12)	7 (14)			
	Secondary	21 (42)	17 (34)			
	Bachelor	21 (42)	21 (42)			
	Higher education	2 (4)	5 (10)			
**Occupation**						.77
	Employed	10 (20)	10 (20)			
	Housewife	22 (44)	23 (46)			
	Retired	18 (36)	16 (32)			
**BMI**						>.99^a^
	Normal weight	14 (28)	13 (26)			
	Overweight	22 (44)	24 (48)			
	Obesity	14 (28)	13 (26)			
Smoking		9 (18)		10 (20)		.80
**Cumulative blood sugar level (%)**						.49^a^
	6-6.9	20 (40)	22 (44)			
	7-7.9	15 (30)	15 (30)			
	8-8.9	5 (10)	8 (16)			
	9-9.9	3 (6)	2 (4)			
	>10	7 (14)	3 (6)			
**Doing exercise**						.88^a^
	Always	1 (2)	1 (2)			
	Frequently	9 (18)	5 (10)			
	Sometimes	7 (14)	14 (28)			
	Rarely	18 (36)	16 (32)			
	Never	15 (30)	14 (28)			
**Medications**						.94
	Insulin	11 (22)	12 (24)			
	Metformin	8 (16)	7 (14)			
	Both medications	1 (2)	2 (4)			
	Others	42 (84)	39 (78)			

^a^Mann-Whitney *U* and chi-square tests were used as appropriate.

Also, about half of the participants were overweight in both groups (44% in the intervention group and 48% in the control group), and the majority were nonsmokers (82% in the intervention group and 80% in the control group). Less than half of participants have 6%-9.9% of blood sugar level (40% in the intervention group and 44% in the control group), and they rarely do exercise (18% in the intervention group and 16% in the control group; Table 1). Also, Mann-Whitney *U* test showed no significant difference between the intervention and control groups in age, BMI, cumulative blood sugar level, educational level, and exercise level at baseline (*P*>.05; Table 1).

### Social Support for Exercise Behavior

The Friedman test was used for changes in social support subdimensions (Table 2). There were no significant differences between baseline, first follow-up, and second follow-up in friends’ support, family practical support, and family emotional support (*P*>.05) in the intervention group. However, there was a significant difference between baseline, first follow-up, and second follow-up in family practical support (*P*<.003) in the control group. This implies less social support as discussed in Table 2. Similarly, repeated measures ANOVA was also used to assess changes in family verbal support differences during the follow-up period for both groups. There were no significant differences between baseline, first follow-up, and second follow-up in family verbal support (*P*>.05) in the intervention group, but were statistically significant in the control group (*P*<.004; Table 2).

**Table 2:** Friedman tests of changes over time in social support for both groups (N=98).

Study group		Intervention group				Control group		
		Mean rank	Statistics	*P* value	Mean rank		Statistics	*P* value
**Friend support^a^**		N/A^b^	1.865	.40	N/A		4.37	.11
	Baseline	1.88			1.84			
	First follow-up	2.02			2.11			
	Second follow-up	2.10			2.05			
**Family practical support^a^**		N/A	1.341	.51	N/A		11.69	<.001
	Baseline	1.92			2.31			
	First follow-up	1.96			1.99			
	Second follow-up	2.12			1.70			
**Family emotional support^a,c^**		N/A	2.191	.33	N/A		2.32	.31
	Baseline	1.98			2.07			
	First follow-up	1.88			2.08			
	Second follow-up	2.14			1.85			

^a^Friedman test was used for changes in social support subdimensions.

^b^N/A: not applicable.

^c^For the family verbal support dimension, repeated measures ANOVA was used. For the intervention group, sum of squares=3.37 (*df*=2), mean sum of squares=1.62, and *F* (*P*)=2.01 (.15); for the control group, sum of squares=4.81 (*df*=2), mean sum of squares=2.41, and *F* (*P*)=5.92 (.004).

### Physical Activity Recall Items

A paired *t* test was used for moderate activities and the Wilcoxon test for hard activities and very hard activities in the intervention group. There was a significant difference (improvement) between the baseline and second follow-up in doing moderate activities in the intervention group (*P*=.03; Table 3). Also, there was a statistically significant difference (improvement) between the baseline and second follow-up in doing very hard activities in the intervention group (*P*<.05; Table 3). However, there was no significant difference between the baseline and second follow-up in challenging activities in the intervention group (Table 3). In regard to the control group, there was a significant difference between the baseline and second follow-up in doing very hard activities (*P*=.01). However, there was no significant difference between baseline and second follow-up in doing moderate activities and challenging activities in the control group (Table 3).

**Table 3:** Differences in various levels of activities over time in both groups.

Activity difficulty levels			Control group					Intervention group						
			Mean		Statistics		*P* value			Mean		Statistics		*P* value
**Moderate activities^a^**			N/A^b^		0.46		.65			N/A		2.28		.03
	Baseline data	1.78							1.82					
	Second follow-up data	1.82							2.08					
**Hard activities^b^**			N/A		0.27		.79			N/A		0.25		.80
	Baseline data	0.55							0.49					
	Second follow-up data	0.55							0.51					
**Very hard activities^c^**			N/A		2.54		.01			N/A		3.20		<.001
	Baseline data	0.12							0.20					
	Second follow-up data	0.11							0.10					

^a^Paired *t* test was used.

^b^N/A: not applicable.

^c^Wilcoxon test was used.

### Effect Size

The mean difference and Cohen *d* value for each subscale were calculated to estimate the effect size of the intervention (Table 4). The effect size of the intervention was small in friends’ support (0.389) and family practical support (0.271), whereas the effect size was medium in family verbal support (0.463) and family emotional support (0.468). In regard to the PA recall scale, the effect size was small in moderate activities (0.386), but no effect in hard activities (−0.093) and very hard activities (−0.082).

**Table 4:** Effect Size for both groups on social support and physical activity subscales.

Domain and study group		Mean differentiation (SD)^a^	Cohen *d*^b^	
**Friend support**				0.389
	Intervention	2.208 (1.24)		
	Control	1.771 (0.99)		
**Family practical support**				0.271
	Intervention	2.680 (1.108)		
	Control	2.395 (0.99)		
**Family verbal support**				0.463
	Intervention	2.827 (1.08)		
	Control	2.362 (0.92)		
**Family emotional support**				0.468
	Intervention	1.750 (0.51)		
	Control	1.528 (0.44)		
**Moderate activities**				0.386
	Intervention	2.077 (0.63)		
	Control	1.814 (0.73)		
**Hard activities**				−0.093
	Intervention	0.508 (0.38)		
	Control	0.543 (0.40)		
**Very hard activities**				−0.082
	Intervention	0.097 (0.15)		
	Control	0.110 (0.16)		

^a^The mean difference.

^b^Cohen *d* value for each subscale was calculated to estimate the effect size of the intervention.

### Coefficients of Social Support for Exercise and Physical Activity Recall Items

Table 5 shows the coefficients of PA recall subscales. Regarding the coefficients of moderate activities model for patients in the intervention group, being a female increased the likelihood of performing moderate activities by 1.6 times (*P=*.002); being married increased the likelihood of doing moderate activities by 1.5 times (*P*=.049) whereas being a housewife decreased the likelihood of doing moderate activities by 20%, (*P=*.001). In regard to the coefficients of the hard activities model for patients in the intervention group, being a female decreased the likelihood of doing hard activities by 20% (*P=*.04), and having a higher educational level decreased the likelihood of doing hard activities by 15% (*P=*.002; Table 5). Regarding the coefficients of very hard activities model, having a higher BMI increased the likelihood of doing very hard activities by 1.06 times (*P=*.02; Table 5).

Table 5 also shows the coefficients of social support for exercise subscales. Regarding coefficients of friends’ support model, being married increased the likelihood of friends’ support by 2.3 times (*P=*.04), whereas rarely doing exercise decreased the likelihood of friends’ support by 28% (*P=*.03). Using coefficients of the family practical support model, rarely doing exercise decreased the likelihood of family practical support by 28% (*P=*.01; Table 5). Regarding coefficients of the family verbal support model as well as family emotional support model, there was no significant association with any of the sociodemographic variables with the 2 subscales.

**Table 5:** The coefficient of physical activity recall and social support for exercise behavior scale.

Model			OR^a^		SE		*t* test (*df*)		*P* value
**Coefficients of moderate activities model^b^**									
	Sex (female)	1.65		0.160		3.13 (48)		.002	
	Marital status	1.50		0.205		1.998 (48)		.049	
	Occupational level	0.80		0.063		−3.39 (48)		.001	
**Coefficients of hard activities model^b^**									
	Sex (female)	0.80		0.098		−2.15 (48)		.04	
	Educational level	0.85		0.051		−3.18 (48)		.002	
**Coefficients of very hard activities model^b^**									
	BMI	1.06		0.025		2.36 (48)		.02	
**Coefficients of friend support model^b^**									
	Marital status	2.31		0.402		2.08 (48)		.04	
	Doing exercise	0.73		0.143		−2.23 (48)		.03	
**Coefficients of family practical support model^b^**									
	Doing exercise	0.72		0.127		−2.53 (48)		.01	

^a^OR: odds ratio.

^b^The coefficients of social support for exercise subscales.

## Discussion

### Overview

This study aimed to assess the effectiveness of a theoretically based distant MMS-based educational intervention on perceived social support and level of PA among patients with T2DM. Overall, there were no significant differences between baseline, first follow-up, and second follow-up in friends’ support, family verbal, practical, or emotional support in the intervention group. However, there was a small effect size on friends’ social support, family practical support, performing moderate activities, and a medium effect size on family verbal and emotional support. Being married increased the likelihood of friends’ support after the intervention, whereas rarely doing exercise decreased the likelihood of friends’ support as well as family’s practical support. Being a female and married in the intervention group increased the likelihood of doing moderate activities. Yet, being a housewife in the intervention group decreased the likelihood of doing moderate activities. Finally, being a female and having a higher educational level decreased the likelihood of doing hard activities.

Regarding the perception of social support from friends and family in PA among patients with diabetes, we did not find any significant improvement in the social support (family or friends) for exercise behavior over time in the intervention group. One possible reason was that the SMS educational approach was not efficient in influencing patients’ friends and family members to provide social support to the patient. These findings support previous literature that patients with type 2 diabetes receive less social support for PA compared with other aspects such as medication and diet for diabetes management [21]. Thus, this should urge activating family, coworkers, and friend’s role to enhance PA and exercise by actively involving them in the intervention process. A study conducted in Iran found improvement in family and friends’ support, which could be due to activating the role of friends and family support by involving them in the education, such as sending them educational messages to motivate them to provide support for their diabetic family member or friend [35]. In fact, if a person has a positive attitude toward a certain health behavior, people around him or her can confirm this behavior and enforce it [36]. This is not uncommon as social support is a vital interpersonal stimulus that defines an individual’s predisposition to engage in health-promoting behaviors [16,17]. Previous studies found that social support from friends, family, and coworkers can result in adopting regular PA [18,19,37]. For patients with T2DM in particular, educating supportive families on self-care management can be beneficial, especially when incorporating exercise social support in educational interventions that focus on improving PA [20-22]. Given the fact that social support alone may not be enough when educating patients with T2DM [23], this study used an intervention that does not only focus on social support but rather incorporates self-efficacy and perceived benefit constructs to ensure a positive change in PA behavior [7].

In measuring the effect of SMS messages on changing exercise behavior among patients with diabetes in the intervention group, we found a significant difference in moderate activities over time with an increase in the mean score between the baseline data and the second follow-up while a decrease in very hard activities. In the control group, we did not find a significant difference in moderate and hard activities, but a decrease in the mean score for very hard activities.

Participants in the intervention group improved their behavior in doing moderate activities over time because they practiced walking on public streets, which is considered moderate activity. Similarly, a study conducted in Jordan [7] reported that patients with diabetes are usually engaged in moderate activities, such as walking or daily activities because this type of activity seems to be safe and convenient. This should alarm both health care providers and policy makers about the need to build safe and accessible public places in which people can perform PA easily without barriers. Also, a national study [7] observed that the factors correlated to physical inactivity were advanced age, higher BMI, more comorbidities, and less exercise self-efficacy. These factors are consistent with our findings, in which females, being a housewife and having sedentary lifestyle behaviors play a vital role in physical inactivity among Jordanian patients with diabetes. It is worth mentioning that the participants in this study were overweight and rarely performed exercise, and this might have decreased noticeable intervention benefits. This is alarming and highlights the need for developing appropriate theoretically based interventions to provide social support to patients to encourage them to perform regular PA.

Additional research that investigates knowledge, attitude, and practice is needed to develop and tailor interventions according to individual patients’ sociodemographic characteristics, needs, and context related to PA. These include the patient’s age, BMI, sex, comorbidities, family and friends’ social support, and patient’s willingness to incorporate PA in their daily lives. This can be best accomplished through feasible, efficient, and affordable tools, such as MMS-based education. Finally, health care providers should personalize PA consultations within routine diabetes primary care services and find the best ways to actively involve family members and friends in PA educational programs.

### Strengths and Limitations

This is the first national study that used MMS through mobile phones to deliver educational interventions to patients with T2DM. The quasi-experimental design and the use of valid and reliable scales were strengths of the study. Additionally, the intervention was developed based on the Health Promotion Model. Nevertheless, this study has some limitations. First, the relatively small number of samples, which was mainly due to the COVID-19 pandemic and that the national lockdown that slowed down the process of recruiting patients. Second, using a convenience sample could have affected the generalizability of the findings; yet, we managed to increase the representativeness of the findings by recruiting all eligible patients who approached the included clinics and voluntarily agreed to participate over the period of data collection.

### Conclusions

The design and implementation of a structured educational program using the Health Promotion Model of PA among patients with T2DM had a positive impact on changes in PA behaviors but not on social support for exercise. Delivering interventions through MMS using cell phones is feasible and cost-effective with a promising impact on adopting healthy lifestyle behaviors, such as PA. Involving family members and friends in PA educational programs for patients with T2DM could yield benefits in social support.

## Abbreviations

MMS: multimedia messaging service
PA: physical activity
T2DM: type 2 diabetes mellitus

## Data Availability

Most of the data generated and analyzed during the study are included in this published paper and others. The remaining collected data are not publicly available due to potential issues with reidentification but may be available from the corresponding author upon reasonable request.
